# MEK inhibition activates STAT signaling to increase breast cancer immunogenicity via MHC-I expression

**DOI:** 10.20517/cdr.2019.109

**Published:** 2020-04-25

**Authors:** Derek A. Franklin, Jamaal L. James, Margaret L. Axelrod, Justin M. Balko

**Affiliations:** 1 Department of Medicine, Vanderbilt University Medical Center, Nashville, TN 37232, USA; 2 Department of Pathology, Microbiology, and Immunology, Vanderbilt University Medical Center, Nashville, TN 37232, USA; 3 Breast Cancer Research Program, Vanderbilt University Medical Center, Nashville, TN 37232, USA

**Keywords:** Mitogen activated protein kinase signaling, immunogenicity, MHC-I, PD-L1

## Abstract

**Aim:**

Immunotherapy and immune checkpoint inhibitors (ICI) have changed cancer care for many patients; however, breast cancers have exhibited minimal response to single agent ICI therapy. There is a significant need to identify novel targets capable of increasing cancer cell immunogenicity and response to ICIs in breast cancer. Mitogen activated protein kinase (MAPK) signaling is essential for many cellular processes but the relationship between MAPK signaling and cancer cell immunogenicity is less well understood. Recent reports suggest that MEK inhibition (MEKi) affects the tumor-immune microenvironment by altering the expression of interferon responsive PD-L1 and MHC-I through unknown mechanisms.

**Methods:**

Using western blotting and flow cytometry, we sought to determine whether MEKi affects JAK-STAT signaling upstream of PD-L1 and MHC-I expression in a panel of mouse mammary cancer and triple negative breast cancer cell lines.

**Results:**

The cell lines tested exhibited increased STAT activation in response to MEKi treatment. Furthermore, MEKi-induced MHC-I and PD-L1 expression are dependent upon STAT1 in MMTV-Neu cells. Interestingly, MEKi-induced STAT activation and interferon-responsive protein expression are abrogated with ErbB-family inhibitor co-treatment in MMTV-Neu cells, suggesting ErbB receptor signaling dependence, but not in basal-like cell lines. Importantly, analysis of basal-like breast cancer patient samples exhibited an inverse relationship between STAT1 and Ras/MAPK activation signatures.

**Conclusion:**

These findings suggest that MAPK signaling and STAT activation are inversely related in both mouse and human mammary tumors. This work also supports further study of MEKi to increase STAT signaling and potentially, immunotherapy responses through increased MHC-I and PD-L1 expression.

## INTRODUCTION

Breast cancer is the most commonly diagnosed malignancy in women with 270,000 new cases expected in 2020^[1]^. Among the clinically defined breast cancer subgroups, triple negative breast cancer (TNBC) is particularly heterogenous and lethal. TNBC is defined by a lack of hormone receptor expression or HER2 amplification. TNBCs respond to chemotherapy initially, but therapeutic resistance and disease progression commonly occur, signifying a need for developing improved therapeutics^[2]^. Studies have found that the presence of tumor-infiltrating lymphocytes are a robust prognostic marker in the response of TNBC to chemotherapy^[3,4]^. Importantly, this finding suggests that immune cells are involved in chemotherapy responses and further, that therapies augmenting tumor immunogenicity may improve outcomes in some TNBCs. Unfortunately, early clinical trial data using immunotherapies such as immune checkpoint inhibitors (ICIs) showed that relatively few patients respond to single agent therapy^[5]^. Recent results from the IMpassion130 trial demonstrated increased progression-free survival for patients with > 1% PD-L1 + immune cells within tumors treated with atezolizumab (α-PD-L1) and nab-paclitaxel compared to nab-paclitaxel alone (25 months *vs.* 15.5 months)^[6]^. This data suggests that PD-L1 expression is important for treatment response to atezolizumab and potentially, other ICIs. Previous work from our laboratory has shown that MEK inhibitor (MEKi) treatment increases PD-L1 and MHC-I expression in two breast cancer lines, and that MEKi/PD-L1 combination treatment inhibits tumor growth compared to either single agent treatment in mice^[7]^. In order to further clarify the relationship between MEKi treatment and PD-L1/MHC-I expression, we need to first determine whether this relationship is conserved in additional breast cancer models, and second, further examine the mechanism by which MEKi treatment induces immune-associated protein expression.

## METHODS

### Cell lines and treatment

Human breast cancer cell lines MDA-MB-231 (DMEM + 10% fetal bovine serum; FBS), HCC1143 (RPMI + 10% FBS), and HCC1954 (RPMI + 10% FBS) were obtained from American Type Culture Collection (ATCC). Murine mammary cancer cell lines 4T1 (DMEM-F12 + 10% FBS) and EMT6 (DMEM-F12 + 10% FBS) were also obtained from ATCC. E0771 cells (RPMI + 10% FBS + 1% HEPES Buffer) were purchased from CH3 Biosystems. MMTV-Neu cells (DMEM-F12 + 10% FBS + EGF 20 ng/mL + Hydrocortisone 0.5 μg/mL + Insulin 10 μg/mL) were derived from a spontaneous tumor within the FVB/N-Tg (MMTV-Neu) 202 Mul/J mouse. All cells were routinely tested for mycoplasma contamination. Cells were treated with 50 nM trametinib (SelleckChem), 1 μM ruxolitinib (SelleckChem), 5 μM itacinib (SelleckChem), 1 μM NVP-BSK805 (provided by Novartis), 0.5 μM erlotinib (SelleckChem), or 0.1 μM lapatinib (SelleckChem).

### siRNAs

Murine cells were reverse transfected using 2.5 μL of 20 μM siRNA stock along with 5 μL of Dharmafect I transfection reagent (Dharmacon) in 500 μL Opti-MEM (Gibco). This was combined with 2 mL of suspended cells in a 6 well culture plate (Corning). Cell lysates were harvested 48 h after transfection, and flow cytometry was performed 4 days after transfection.

Non-targeting pool (siNTC): UGGUUUACAUGUCGACUAA, UGGUUUACAUGUUGUGUGA, UGGUUUACAUGUUUUCUGA, UGUUUACAUGUUUUCCUA

siSTAT1 (mouse): GGAUUUCGGAAGUUCAACATT, UGUUGAACUUCCGAAAUCCTT

siSTAT3 (mouse): GAGUUGAAUUAUCAGCUUATT, UAAGCUGAUAAUUCAACUCAG

siSTAT5a (mouse): GACGCGAGAUUUCUCCAUUTT, AAUGGAGAAAUCUCGCGUCGT

### Immunoblotting

Immunoblotting was performed as previously described^[8]^. Briefly, tumor fragments were homogenized in 1× RIPA buffer (0.1% SDS detergent, 50 mM Tris pH 7.4, 150 mM NaCl, 1.0% NP-40, 0.5% deoxycholic acid, 1 mM EDTA, 1 mM EGTA, 5 mM sodium pyrophosphate, 50 mM NaF, 10 mM b-glycerophosphate) with added phosphatase inhibitors (PhosSTOP, Roche) and protease inhibitors (cOmplete, Roche). Lysates were incubated on ice for 15 min before centrifugation at 13,000 × *g* for 15 min at 4 °C. Protein concentrations of the lysates were determined by BCA assay (Thermo). Samples were separated on NuPage 4%-12% BisTris gels (Invitrogen) and transferred to nitrocellulose membranes. Membranes were blocked with 5% non-fat dry milk or 5% BSA in tris-buffered saline (TBS) with 0.1% Tween-20 for 1 h at room temperature and then incubated overnight at 4 °c with the appropriate antibody in blocking buffer as indicated. Following incubation with appropriate horseradish peroxidase-conjugated secondary antibodies, proteins were visualized using an enhanced chemiluminescence detection system (Thermo). This study was performed using the following antibodies: calnexin (#SC11397; Santa Cruz), STAT1 (#SC-592, Santa Cruz), STAT5a (SC-1081, Santa Cruz), ERK1/2 (#9102), p-ERK1/2 (#4370), pY-STAT1 (#7649), pY-STAT3 (#9145), STAT3 (#9139) and pY-STATs (#9359), all of which were purchased from Cell Signaling Technologies.

### Flow cytometry

Cells were washed in phosphate-buffered saline (PBS) and harvested with Accutase (EMD Millipore, #SCR005) for 10 min at room temperature. Dissociated cells were washed once in flow staining buffer (PBS + 1% FBS) and incubated with respective flow antibodies at 4 °c for 20 min in the dark. Flow cytometry was performed using the following antibodies: H2Kq/AF647 (Biolegend clone KH114, 1:200), PD-L1/PE (BioLegend Clone 10F.9G2, 1:100), H2Kb/AF488 (BioLegend Clone AF6-88.5, 1:400), H2Kb-SIINFEKL (BioLegend Clone 25-D1.16, 1:200), H2Kd/PE (BioLegend Clone SF1-1.1, 1:400). DAPI was used as a viability dye for dead cell exclusion. Samples were analyzed on an Attune NxT flow cytometer (Life Technologies).

### TCGA transcriptional analysis

Gene expression data for the 50-gene IRDS signature were extracted from TCGA breast^[9]^ “Provisional” dataset and associated clinical metadata were accessed via the cBio portal^[10]^. Molecular subtype analysis was performed using the genefu package^[11]^ in R^[12]^. The 50-gene MEK transcriptional signature was calculated as previously described^[7,13]^. The STAT1 signature score was derived as a 50 gene signature from previous studies^[14,15]^ and was calculated by summing the normalized log_2_ Z-scores of the expression data from the 50 genes.

## RESULTS

**Figure 1.**
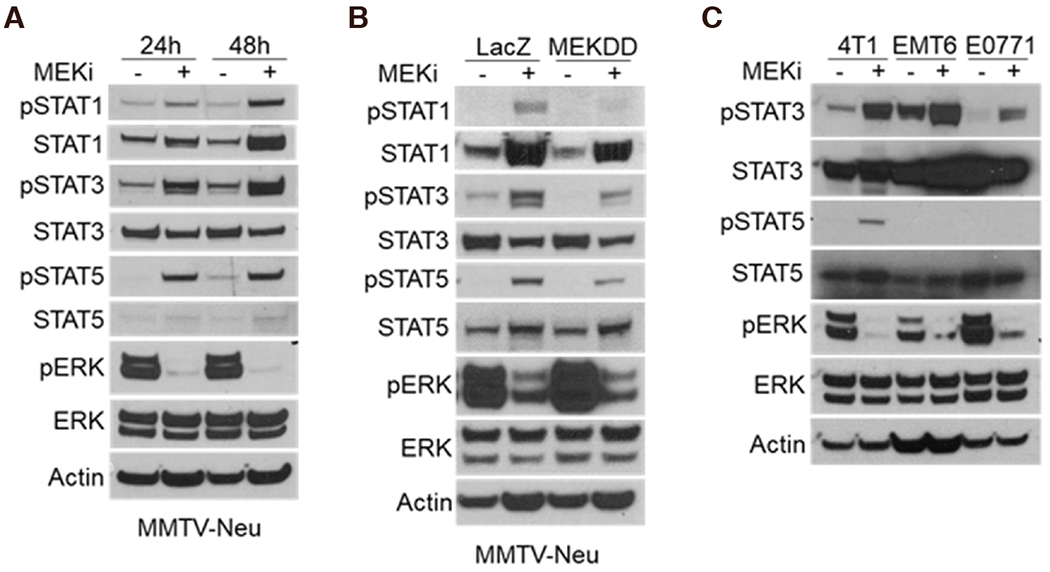
Ras/MAPK activity is inversely related to STAT activation in murine mammary cancer cell lines. A: immunoblot analysis of MMTV-Neu cells treated with or without trametinib (50 nM; MEKi) for 24 or 48 h prior to lysis; B: MMTV-Neu cells stably expressing *LacZ* or constitutively active*MEK^DD^* were treated and analyzed as in A; C: 4T1, EMT6, and E0771 murine mammary cells were treated and analyzed as in A. MAPK: mitogen activated protein kinase; MEKi: MEK inhibition

Reports have shown that activation of the JAK/STAT pathway leads to increased expression of PD-L1 and MHC-I^[16]^. Other studies have indicated that mitogen activated protein kinase (MAPK) signaling affects JAK/STAT activation in certain contexts, but whether this relationship is conserved in breast tumors is unknown^[17–19]^. In order to determine whether STAT activation occurs in response to MEKi treatment, MMTV-Neu mammary cancer cells were treated with MEKi prior to phospho-tyrosine (pSTAT) and total protein (STAT) evaluation via western blotting. There are two isoforms of STATs denoted as STAT5a and STAT5b; however, the pSTAT5 antibody used in this work recognizes phosphorylation of both isoforms. We observed consistent activation of STAT1, STAT3, and STAT5 in response to MEKi [Figure 1A]. Moreover, MMTV-Neu cells expressing a constitutively active *MEK^DD^* allele exhibited increased ERK activation and decreased STAT activation compared to control *LacZ*-expressing cells [Figure 1B]. To determine whether STAT activation is inversely related to ERK activity in additional mammary cancer models, a panel of murine mammary cancer cell lines were treated with MEKi. While basal pSTAT levels varied between 4T1, EMT6, and E0771 cells, each line exhibited increased pSTAT3 in response to MEKi whereas only 4T1 cells exhibited a detectable level of pSTAT5 in response to MEKi treatment [Figure 1C]. Taken together, MEKi treatment broadly induced STAT activation in multiple murine mammary cancer cell lines.

**Figure 2.**
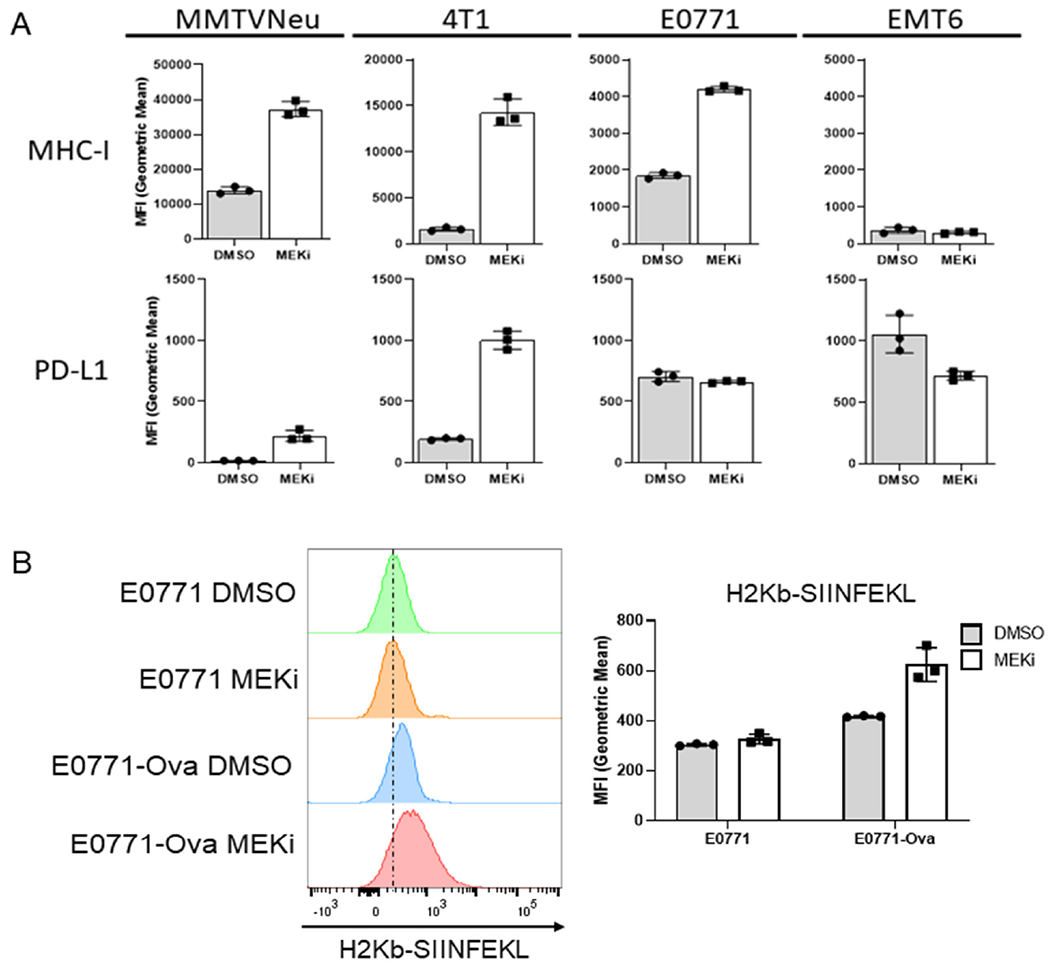
MEKi treatment increases immue-associated protein expression and antigen presentation in murine mammary cancer cell lines. A: MMTV-Neu, 4T1, EMT6, and E0771 murine mammary cells were treated with or without trametinib (50 nM; MEKi) for 72 h prior to flow cytometry analysis for MHC-I and PD-L1 expression; (*n* = 3); B: E0771 and E0771-Ova cells were treated with or without trametinib (50 nM; MEKi) for 72 h prior to flow cytometry analysis for MHC-I (H2Kb), MHC-I bound to SIINFEKL (H2KB-SIINFEKL) and PD-L1 expression. (*n* = 3). MEKi: MEK inhibition; DMSO: dimethylsulfoxide

We have previously shown that MEKi treatment synergizes with IFN-γ to induce interferon-responsive MHC-I and PD-L1 expression in certain murine mammary cancer cell lines, but with only modest effects observed for MEKi treatment in the absence of IFN-γ^[7]^. Given the STAT activation observed in response to MEKi treatment, we sought to determine whether MEKi treatment also increases MHC-I and PD-L1 expression. MMTV-Neu and 4T1 cells exhibited significant increases in both MHC-I and PD-L1 expression at 72 h post MEKi treatment [Figure 2A]. E0771 cells strongly increased MHC-I expression with no significant change observed in PD-L1 expression [Figure 2A]. Surprisingly, EMT6 cells exhibited relatively high basal levels of PD-L1 but no significant change in PD-L1 or MHC-I expression in response to MEKi treatment [Figure 2A]. Therefore, MEKi treatment induces MHC-I and PD-L1 expression changes in most, but not all murine mammary cancer lines that were evaluated. In order to determine whether increased MHC-I expression affects tumor cell autonomous antigen presentation, we treated transferrin receptor-ovalbumin expressing E0771 (E0771-Ova) cells with MEKi prior to flow analysis using antibodies specific for total MHC-I (H2Kb) and MHC-I presenting the class-I-restricted ovalbumin antigen SIINFEKL (H2Kb-SIINFEKL)^[20,21]^.

Importantly, E0771-Ova cells treated with MEKi exhibited increased H2Kb-SIINFEKL staining, which suggests that MEKi treatment upregulates functional antigen presentation by MHC-I in E0771 cells [Figure 2B].

**Figure 3.**
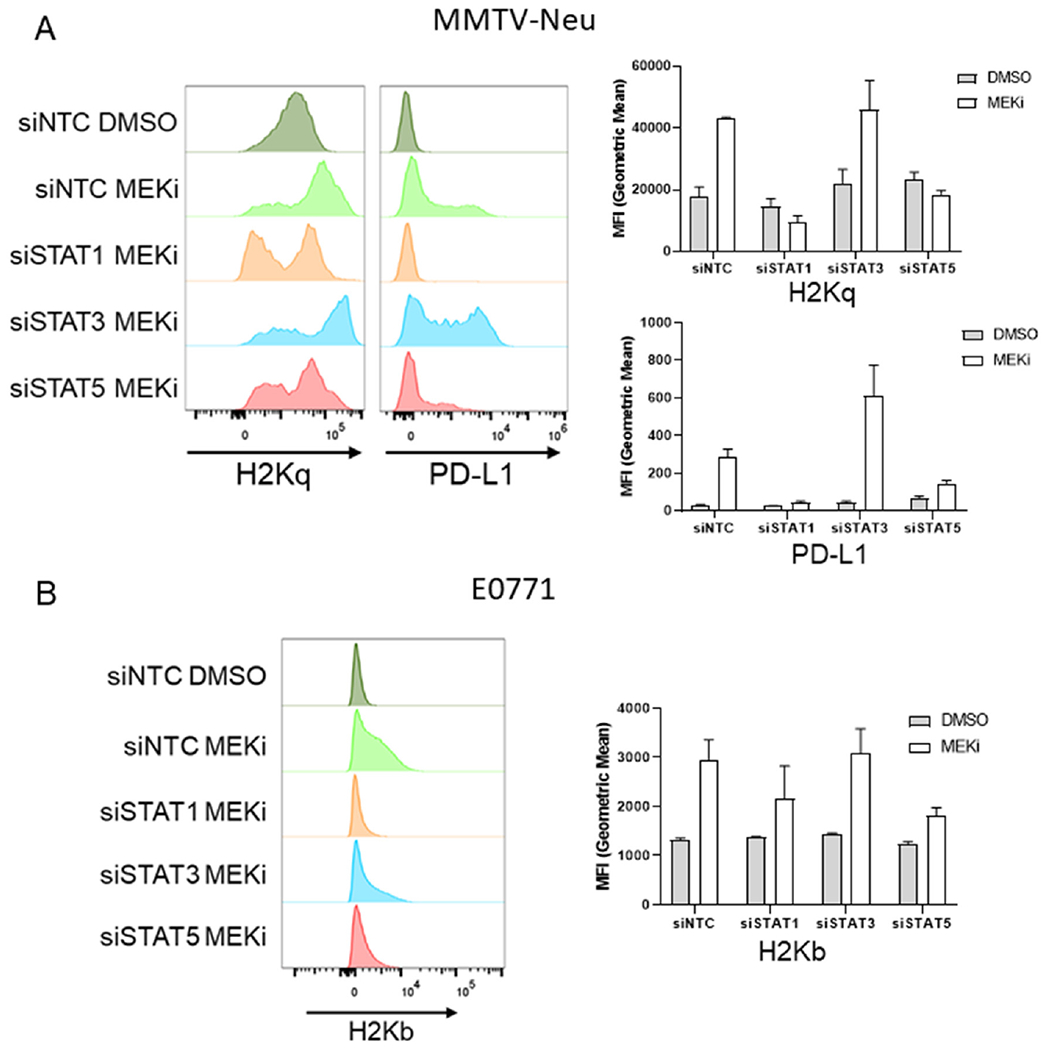
MEKi-induced MHC-I & PD-L1 expression are STAT dependent in MMTV-Neu and E0771 cells. A: MMTV-Neu cells were reverse transfected with siRNAs against a non-targeting control (siNTC), STAT1, STAT3, or STAT5a prior to treatment with or without trametinib (50 nM; MEKi) for 72 h prior to flow cytometry analysis for MHC-I (H2Kq) and PD-L1 expression; (*n* = 3); B: E0771 cells were similarly transfected and treated as in A prior to flow cytometry analysis for MHC-I (H2Kb). MEKi: MEK inhibition; DMSO: dimethylsulfoxide

In order to determine whether MEKi-induced PD-L1 and MHC-I are STAT dependent, MMTV-Neu cells were treated with siRNAs targeting STAT1/3/5a or a non-targeting control prior to vehicle or MEKi [Supplemental Figure 1]. Interestingly, MMTV-Neu MHC-I and PD-L1 expression levels were strongly down-regulated in the siSTAT1 treated samples, and to a lesser degree in the siSTAT5a samples [Figure 3A]. A similar trend was observed for MHC-I expression in E0771 cells co-treated with siSTAT1/5 and MEKi, which suggests a similar dependence upon STAT1/5 activation [Figure 3B]. Importantly, STAT knockdowns did not affect basal MHC-I or PD-L1 expression in either MMTV-Neu or E0771 cells [Supplemental Figure 2].

**Figure 4.**
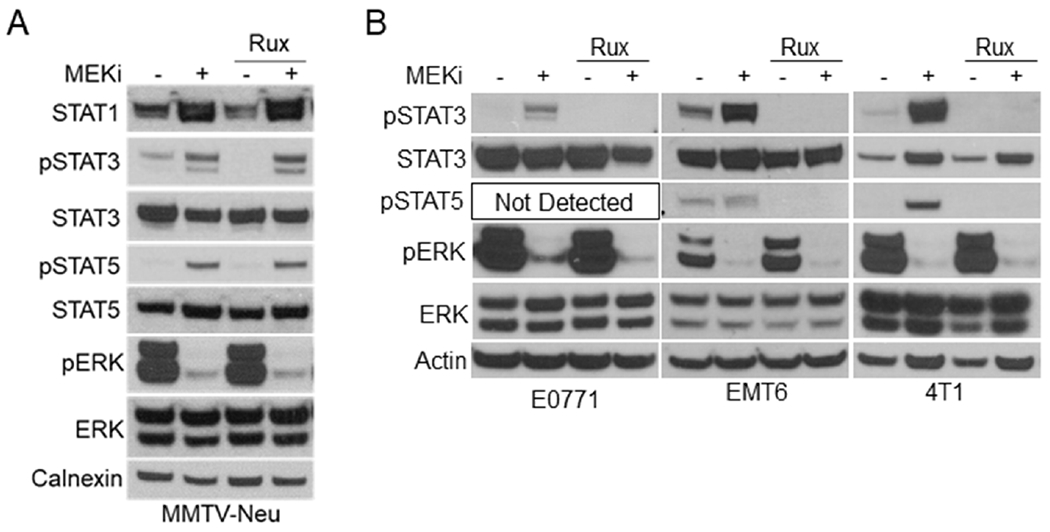
MMTV-Neu cells exhibit insensitivity to JAK inhibitors compared to other murine mammary cancer cell lines. A: immunoblot analysis of MMTV-Neu cells treated with or without trametinib (50 nM; MEKi) and/or Ruxolitinib (1 μM; Rux) for 48 h prior to lysis; B: immunoblot analysis of E0771, EMT6, and 4T1 cells treated as in A. MEKi: MEK inhibition

Previous reports have suggested a direct link between MEK-ERK and Janus kinases (JAK) upstream of STAT activation in various cancer cells^[14,22]^. We initially tested a panel of JAK inhibitors including the JAK1/2 inhibitor ruxolitinib, JAK1-specific itacinib, and JAK2-specific BSK805 [Figure 4A; Supplemental Figure 3]. Interestingly, JAK inhibitors decreased basal STAT1 expression and STAT3 activation levels compared to vehicle treated controls but exhibited no effect on MEKi-induced STAT activation in MMTV-Neu cells [Figure 4A]. Conversely, ruxolitinib co-treatment with MEKi reduced STAT3 activation in E0771, 4T1, and EMT6 cell lines [Figure 4B]. Importantly, STAT5 activation was also inhibited by ruxolitinib in cell lines where activation of STAT5 was detectable. These data suggest that MEKi-induced activation of STAT3/5 utilize different mechanisms in MMTV-Neu cells compared to other murine mammary cancer cell lines.

**Figure 5.**
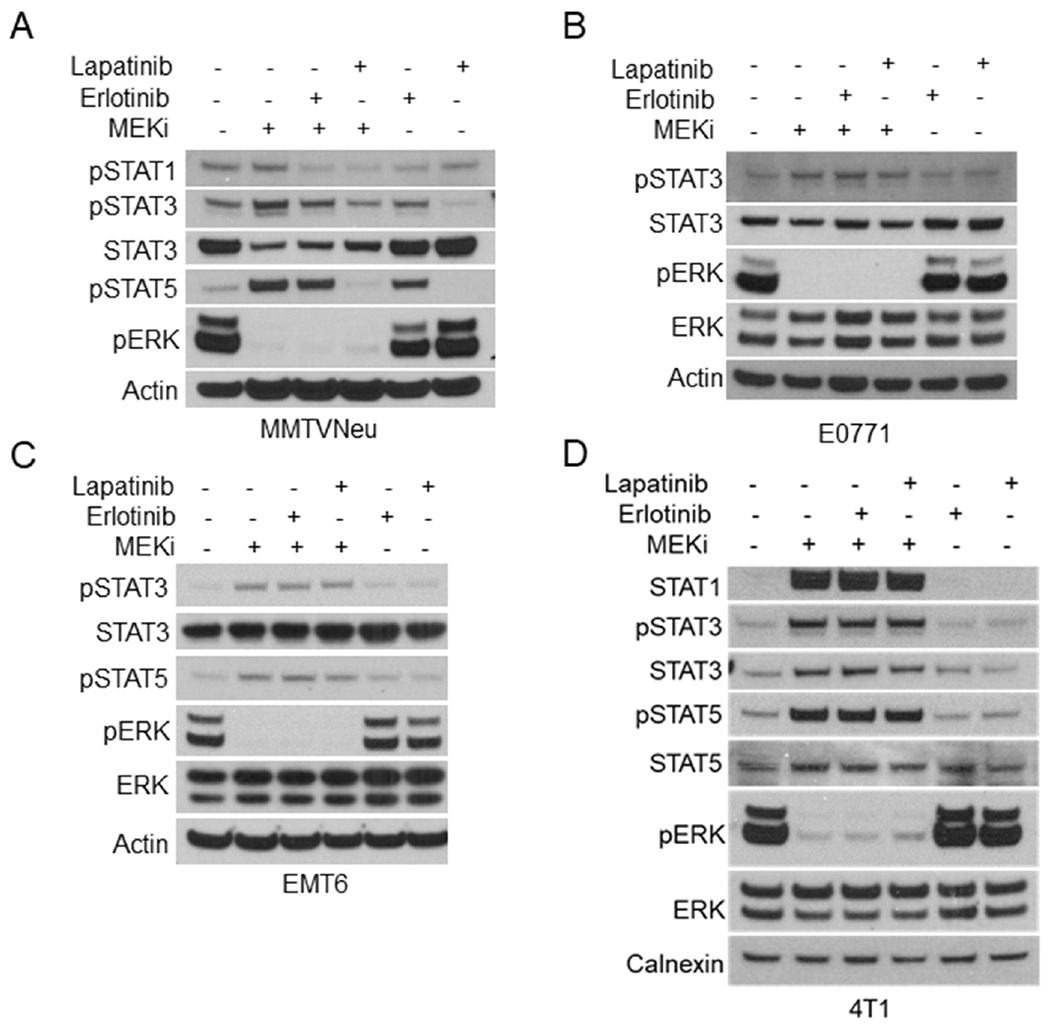
ErbB family signaling bypasses JAK activation in MMTV-Neu cells. A-D: immunoblot analysis of MMTV-Neu (A), E0771 (B), EMT6 (C), and 4T1 (D) cells treated with or without trametinib (50 nM; MEKi) along with Erlotinib (0.5 μM) or Lapatinib (0.1 μM) for 48 h prior to lysis. MEKi: MEK inhibition

**Figure 6.**
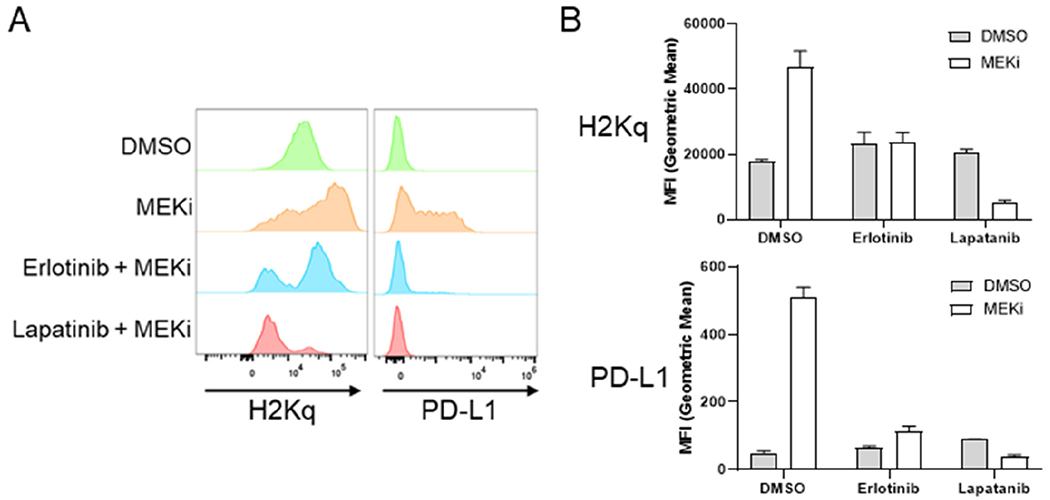
MEKi-induced MHC-I & PD-L1 expression are ErbB family kinase dependent in MMTV-Neu cells. MMTV-Neu murine mammary cells were treated with or without trametinib (50 nM; MEKi) and/or Erlotinib (0.5 μM) or Lapatinib (0.1 μM) for 72 h prior to flow cytometry analysis for MHC-I (H2Kq) and PD-L1 expression. Histogram plots (A) and bar graphs of MFI (B) (*n* = 3). MEKi: MEK inhibition; DMSO: dimethylsulfoxide

One potential explanation for the variation between the other murine mammary cancer cell lines and MMTV-Neu cells is the expression of rat Neu (ErbB2/Her2) receptor^[23]^. In order to evaluate whether ErbB2 activity affects MEKi-treatment induced STAT activation, we co-treated MMTV-Neu cells with MEKi and erlotinib (EGFR/ErbB1 inhibitor) or MEKi and lapatinib (ErbB1 and ErbB2/Neu inhibitor). Erlotinib co-treatment modestly inhibited STAT activation; however, lapatinib co-treatment completely ablated MEKi-induced STAT activation to basal levels, which suggests that STAT activation in response to MEKi treatment is dependent upon ErbB2 receptor activity [Figure 5A]. Importantly, neither erlotinib or lapatinib co-treatment affected MEKi-induced STAT activation in 4T1, EMT6, or E0771 cells [Figure 5B–D]. Next, we tested whether inhibition of ErbB family receptors would affect MHC-I and PD-L1 expression. Basal MHC-I and PD-L1 expression levels were unaffected by erlotinib or lapatinib treatment [Supplemental Figure 4]. Importantly, PD-L1 and MHC-I expression were significantly reduced in MMTV-Neu cells co-treated with MEKi and erlotinib [Figure 6A and B]. Similar to the effects of lapatinib co-treatment upon STAT activation, MEKi-lapatinib combination treatment completely ablated MHC-I and PD-L1 expression, which further suggests that MEKi induces MHC-I and PD-L1 expression in a STAT and ErbB family receptor dependent manner [Figure 6A and B].

**Figure 7.**
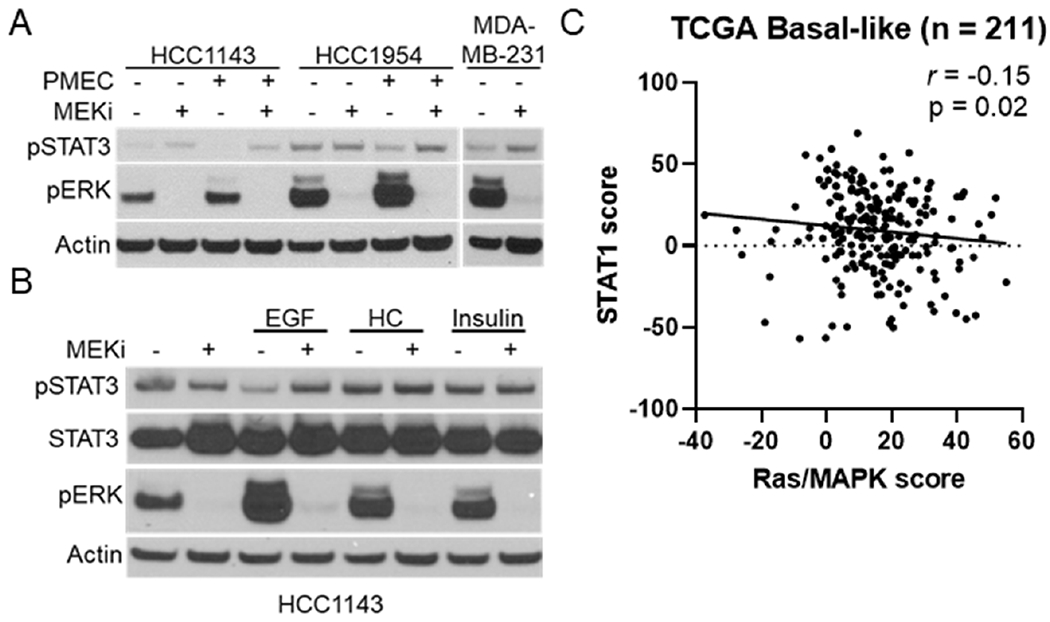
MAPK signaling and STAT activation are inversely correlated in human TNBC cell lines and basal-like patient samples. A: immunoblot analysis of HCC1143, HCC1954, and MDA-MB-231 cells grown in either base media or PMEC media treated with or without trametinib (50 nM; MEKi) for 48 h prior to lysis; B: immunoblot analysis of HCC1143 cells grown in RPMI media supplemented with EGF (20 ng/mL), hydrocortisone (0.5 μg/mL), or insulin (10 μg/mL) along with being treated with or without trametinib (50 nM; MEKi) for 48 h prior to lysis; C: transcriptional analysis of STAT1 and Ras/MAPK scores for 211 Basal-like breast cancers. MAPK: mitogen activated protein kinase; TNBC: triple negative breast cancer; MEKi: MEK inhibition

Previous reports have suggested a connection between MEK-ERK and STAT signaling in pancreatic cancer patient-derived xenograft models, but whether the relationship is conserved in breast cancers is unknown^[24]^. We initially tested a panel of human breast cancer cell lines for MEKi-induced STAT activation [Figure 7A; Supplemental Figure 5]. Surprisingly, only the HCC1143 and MDA-MB-231 cell lines exhibited clear STAT3 activation in response to MEKi treatment. As MMTV-Neu cells exhibited the strongest STAT activation and are grown in DMEM-F12 media supplemented with FBS, EGF, hydrocortisone, and insulin (PMEC), we sought to determine whether human cell lines may activate STAT3/5 in response to MEKi in the presence of these conditions. Indeed, HCC1143 and HCC1954 cells exhibited increased STAT3 activation in response to MEKi in PMEC media [Figure 7A]. Further experiments on HCC1143 cells demonstrated that EGF-mediated increases in MAPK activation reduced basal STAT3 activation in a MEK dependent manner, strengthening the connection between MAPK signaling and STAT3 activation in human breast cancer cell lines [Figure 7B]. To determine whether this connection could be observed in human breast tumors, we probed the TCGA basal-like breast cancer data set for a previously published Ras/MAPK activity score and a STAT1 activation score^[7,17–20]^. Across 211 basal-like breast cancers, a statistically significant negative correlation was observed between Ras/MAPK and STAT1 activation [Figure 7C]. Thus, these data show that Ras/MAPK and STAT1 signaling are inversely related in human breast cancers.

## DISCUSSION

The data presented here show that inhibition of MAPK signaling via MEKi treatment increases surface expression of MHC-I and PD-L1 in murine mammary cancer cell lines via STAT activation. A similar relationship between MAPK pathway activation and STAT signaling was also detected in human breast cancer cell lines and TCGA patient data, suggesting conservation of the MEK-ERK-STAT pathway. Furthermore, MEKi pretreatment led to a functional increase in E0771 tumor cell immunogenicity observed via increased presentation of the model antigen SIINFEKL derived from ovalbumin. Taken together, these findings suggest that MEKi could be used to boost the antigen presentation of breast tumors prior to T cell targeting therapies such as ICIs. Currently, ICIs are only approved for PD-L1 positive immune infiltrated TNBCs in combination with the chemotherapeutic nab-paclitaxel; however, increased tumor immunogenicity via MEKi pretreatment could lead to increased ICI efficacy in TNBCs. Moreover, further work is needed to determine whether the MEKi-induced immunogenicity observed in MMTV-Neu cells is conserved in other HER2 amplified breast cancer cell lines. Consequently, MEKi could improve ICI response and expand ICI therapy to treatment resistant HER2-amplified cancers.

Previous studies have shown that MEKi treatment inhibits naïve T cell activation and this could be bypassed via cotreatment with a T cell agonist such as α-OX-40^[26]^. Determining whether MEKi pretreatment prior to T cell targeted therapies can also bypass T cell inhibition will require further study. Moreover, it is unknown whether MEKi-induced PD-L1 expression prognosticates ICI efficacy in the same manner as basal PD-L1 expression levels do. Accordingly, MEKi pretreatment could expand the use of anti-PD-L1/nab-paclitaxel to TNBCs initially exhibiting low PD-L1 levels.

The focus of this work has been to assess the effect of MEKi treatment on the immunogenicity of tumors via antigen presentation and response to immunotherapy prognosticated by PD-L1 expression. MEKi treatment could also lead to increased immunogenic cell death for cancers that have developed dependence upon MAPK signaling. Primary breast cancers exhibit relatively low levels of canonical Ras/MAPK alterations, but these alterations have been shown to be increased in metastatic tumors and ER+ tumors in which therapeutic resistance has developed, suggesting additional populations in which immunogenic breast cancer cell death induced by MEKi treatment will require further investigation^[27]^.
