# Supplementary material for: MEK inhibition activates STAT signaling to increase breast cancer immunogenicity via MHC-I expression
Source: Cancer Drug Resist. 2020 Apr 25;3(3):603–12. doi: 10.20517/cdr.2019.109 (PMC7556720; doi:10.20517/cdr.2019.109)
Supplement: Supplementary file 1 [file cdr-3-603-SupplementaryMaterials.pdf]

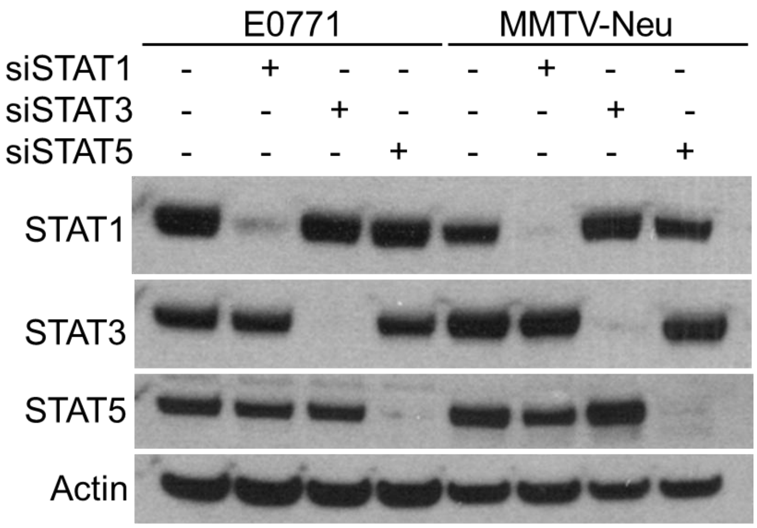

Supplemental Figure 1. siSTAT treatment depletes E0771 and MMTV-Neu STAT expression. Immunoblot analysis STAT expression in E0771 and MMTV-Neu cells 48h after siRNA transfection.

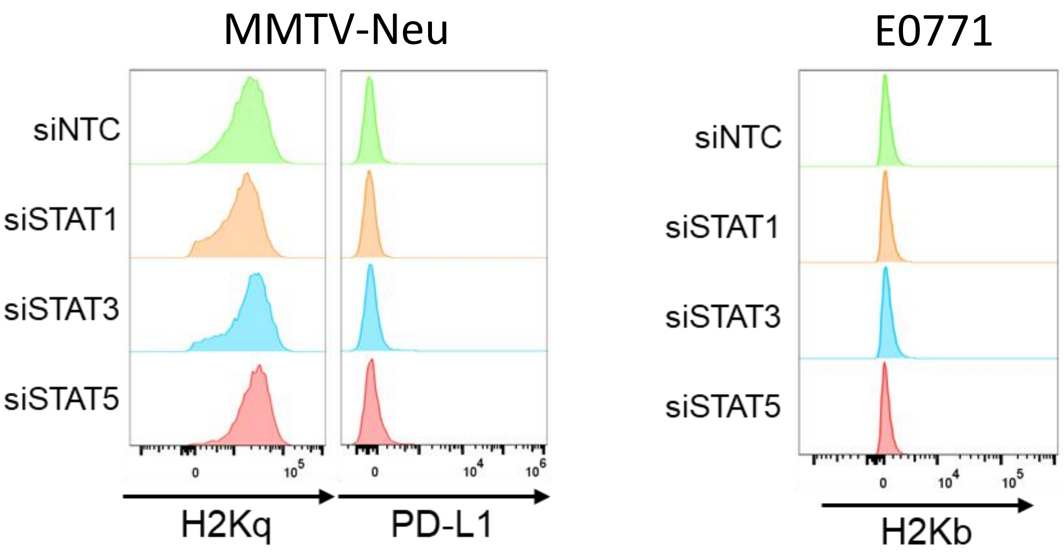

Supplemental Figure 2. Basal expression of MHC-I and PD-L1 are unchanged in response to siSTAT treatment in MMTV-Neu and E0771 cells. Flow cytometry analysis of vehicle treated MMTV-Neu and E0771 cells 96h after transfection.

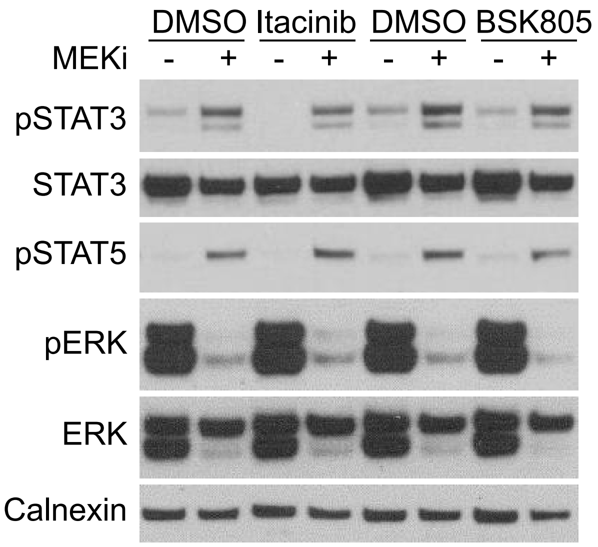

Supplemental Figure 3. MMTV-Neu cells exhibit insensitivity to JAK1/2 specific inhibitors. Immunoblot analysis of STAT activation in MMTV-Neu cells treated with or without MEKi and itacinib (5 μM) or BSK805 (1 μM) for 48h.

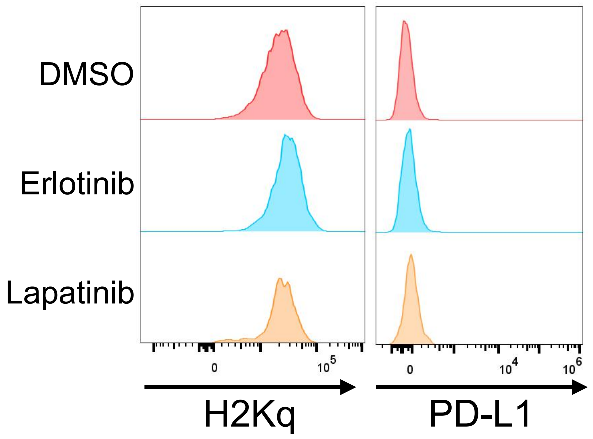

Supplemental Figure 4. Basal expression of MHC-I and PD-L1 are unchanged in response to ErbB family kinase inhibitors in MMTV-Neu cells. Flow cytometry analysis of vehicle, erlotinib, or lapatinib treated MMTV-Neu cells after 72h.

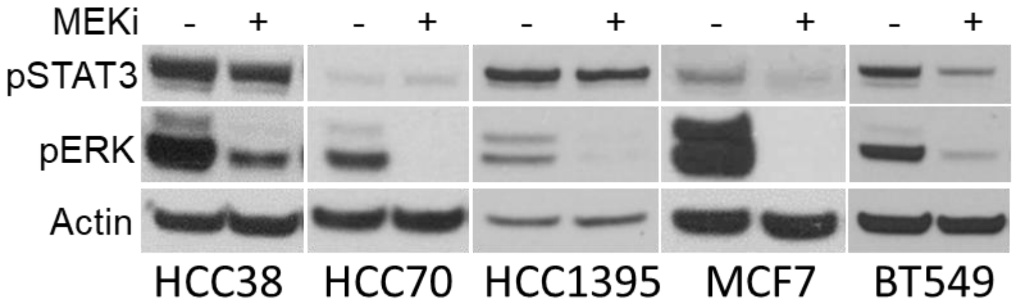

Supplemental Figure 5. Human breast cancer cells exhibit varied STAT3 responses after MEK inhibitor treatment. Immunoblot analysis of STAT3 and ERK activation after treatment with or without MEKi for 48h.
